# Structure and Antioxidant Activity of Polyphenols Derived from Propolis

**DOI:** 10.3390/molecules19010078

**Published:** 2013-12-20

**Authors:** Anna Kurek-Górecka, Anna Rzepecka-Stojko, Michał Górecki, Jerzy Stojko, Marian Sosada, Grażyna Świerczek-Zięba

**Affiliations:** 1 Silesian Medical College in Katowice, Mickiewicza 29, Katowice 40-085, Poland; E-Mail: grazyna.swierczek_zieba@swsm.pl; 2 Department of Pharmaceutical Chemistry, School of Pharmacy with the Division of Laboratory Medicine, Medical University of Silesia, Jagiellońska 4, Sosnowiec 41-200, Poland; E-Mail: annastojko@sum.edu.pl; 3 Department of Drug Technology, School of Pharmacy with the Division of Laboratory Medicine, Medical University of Silesia, Jedności 8, Sosnowiec 41-200, Poland; E-Mails: mgorecki@sum.edu.pl (M.G.); msosada@sum.edu.pl (M.S.); 4 Department of Hygiene, Bioanalysis and Environmental Studies, School of Pharmacy with the Division of Laboratory Medicine, Medical University of Silesia, Kasztanowa 3A, Sosnowiec 41-200, Poland; E-Mail: jstojko@sum.edu.pl

**Keywords:** propolis, antioxidant activity, phenolic acids, flavonoids

## Abstract

Propolis is a potential source of natural antioxidants such as phenolic acids and flavonoids. Its wide biological effects have been known and used since antiquity. In the modern world natural substances are sought which would be able to counteract the effects of antioxidative stress, which underlies many diseases, such as cancer, diabetes and atherosclerosis. This paper aims to present the antioxidative activity of phenolic acids and flavonoids present in Polish propolis and the relationship between their chemical structure and antioxidative activity influencing its medicinal properties. Data concerning the biological activity of propolis are summarized here, including its antibacterial, anti-inflammatory, anticarcinogenic, antiatherogenic, estrogenic effects, as well as AIDS- counteracting and reparative-regenerative function.

## 1. Introduction

Propolis belongs to the products collected and processed by bees. Propolis is a plant product—a resinous mixture collected by bees from deciduous tree buds and crevices in the bark of coniferous and deciduous trees, to which beeswax and the secretion of bee mandibular and hypopharyngeal glands are added. Apart from resins, constituting about 50% of the propolis, and wax, constituting about 30% of its content, propolis also contains essential oils, pollen and other organic components, constituting respectively 10%, 5% and 5% of its content [1,2,3]. The content of biologically active substances in Polish propolis may be up to 70%. Polyphenolic compounds comprise 58% of this amount. Out of this 58%, 20% are flavonoids [4].

Analyses of numerous propolis samples from different geographical zones display great differences in the composition of propolis, which in turn influences its biological activity. However, despite the diversity of components in the propolis coming from various parts of the World, it always displays high antibacterial, antiviral, antioxidative, antifungal and antiatherogenic, as well as antiproliferative and proapoptotic activity. Some varieties of propolis may display an increased antiproliferative and anti-inflammatory activity, regenerative-reparative, estrogenic and anesthetizing properties, as well as proapoptotic activity towards cancer cells. Propolis also displays cardioprotective and hepatoprotective properties [3,5,6,7,8]. Due to such a wide spectrum of activity, research into domestic propolis is essential. In Poland, just as in the countries of Central and Eastern Europe, most propolis is collected by bees from black poplar (*Populus nigra* L.), birch (*Betula pendula* Roth), alder (*Alnus* *glutinosa* L.), pine (*Pinus sylvestris* L.) and willow species (*Salix sp.* L.) buds [1,9]. Therefore, many researchers qualify Polish propolis as a poplar type propolis, because it contains components typical for the European ‘poplar bud’ propolis - phenolics: flavonoid aglycones, phenolic acids and their esters [8,10]. Because the complex chemical composition of propolis depends on the area from which it is derived, as well as on the breed of bees, new chemical compounds are sometimes identified among its components, which can influence its biological properties [10]. The high medicinal activity and therapeutic effectiveness of propolis has contributed to its wide use in traditional medical practice. Raw propolis is rarely used, but rather condensed Ethanolic Extract of Propolis (EEP), obtained by extraction with 70% ethanol, is used for medical, dietetic and cosmetic purposes. The antioxidative activity of EEP, resulting from the presence of phenolic components, deserves attention. 

## 2. Propolis Composition

So far, some 300 compounds have been identified in propolis. The following can be discerned as the basic groups among these compounds: phenolic acids, flavonoids, terpenes, lipid-wax substances, beeswax, bioelements and other components such as vitamins, proteins, amino acids and sugars [4,8,11]. 

Phenolic compounds constitute the most numerous group of propolis components with respect to the quantity and type. Among them there are phenolic acids, phenolic aldehydes, phenols and their esters, ketophenols, coumarins and others compounds, including eugenol, anethole, hydroquinone, pterostilbene, naphthalene, *etc*. [3,4,12]. According to Polish researchers, the content of polyphenolic compounds in domestic propolis amounts to about 58%, while in the condensed extract it is 78% [13,14].

Phenolic acids in the poplar type propolis are represented mainly by benzoic and cinnamic acid, together with their derivatives. Among the most common derivatives of benzoic acid are *p*-hydroxy-benzoic acid, *p*-methoxybenzoic acid (also called anisic acid) and gallic acid. Furthermore, protocatechuic acid, salicylic acid, gentisic acid, 3,4-dimethoxybenzoic acid, vanillic acid and 2-amino-3-methoxybenzoic acid are present [1,4]. 

Among the cinnamic acid derivatives most often present in Polish propolis there are caffeic acid, ferulic acid, *p*-coumaric acid, isoferulic acid and 3,4-dimethoxycinnamic acid. Apart from the above, the following are present: *o*-coumaric acid, *m*-coumaric acid, coumarinic acid, sinapic acid, hydrocaffeic acid, and cinnamylideneacetic acid [14,15]. In 2002 Maciejewicz *et al.* [16] identified 4-hydroxybutyric acid (4-hydroxybutanoic acid), 3-hydroxybutyric acid (3-hydroxybutanoic acid) for the first time, as well as phthalic acid, 4-hydroxyhydrocinnamic acid (phenyllactic acid), imidazole and phenetole in samples of propolis from the southern regions of Poland. 

Phenol alcohols present in propolis include benzyl and cinnamyl alcohol, as well as coniferyl alcohol, coumaryl alcohol and cyclohexanol. Phenol alcohols produce esters, which are numerous in Polish propolis. The most important aromatic esters are as follows: benzoic acid benzyl ester, cinnamic and caffeic acid ether ester and benzoic acid phenylmethyl ester [1].

Acetophenone and methylacetophenone are the ketophenols discovered in propolis. Among phenolic aldehydes there are vanillin and isovanillin. Frequently, benzoic, cinnamic, *p*-coumaric and coniferyl aldehyde are identified [4]. 

Apart from phenolic compounds, phenylvinyl and phenyl-p-methoxyvinyl ether are also found in propolis, as well as coumarins like coumarin and daphnetin [5,6]. 

Among aromatic ethers in propolis the following dominate: cinnamic acid ethyl ester, benzoic acid ethyl ester, salicylic acid phenylmethyl ester, and benzoic acid phenylmethyl ester [1].

Another group of chemical compounds present in European propolis are flavonoids. These are substances with multiphenol character. Dobrowolski *et al.* discovered as many as 38 flavonoids in propolis [17]. Flavonoids present in propolis are aglycones of glycosidic substances present in plants. While collecting propolis, bees secrete β-glucosidase, which hydrolyzes glycosides of flavonoids to the corresponding aglycones and sugars [13,18].

EEP contains on average six to nine flavonoid compounds [1,17]. Maciejewicz *et al.* identified nine flavonoids in Polish propolis: tectochrysin, pinocembrin, chrysin, galangin, genkwanin, apigenin, kaempferol, and 5-hydroxy-4',7-dimethoxyflavone. They also isolated pilloin and pinostrobin chalcone for the first time [19]. 

Aside from the two basic groups of chemical compounds—phenolic acids and flavonoids—based on the content of which it is possible to classify a given propolis as poplar type, there are also terpenes present in it. The content of these compounds in propolis is usually small—it amounts to about 0.5%. Among the terpenes isolated from propolis are monoterpenes, sesquiterpenes and triterpenes. Monoterpenes are represented by geraniol, nerol and borneol, sesquiterpenes are represented by β-eudesmol, α-acetoxybetulenol, β-bisabolol, kariofilen, guaiol, guaiene, β-selinene and farnesol, while the triterpens are squalene and glutinol [10,15,20].

Furthermore, there is a group of substances present in propolis called plant waxes or lipid-wax substances. Plant waxes are composed of sterols, fatty acids and their esters, especially phenolic acids and glycerol esters [21]. Maciejewicz *et al.* isolated stigmasterol, cholinasterol, fucosterol and dihydrofucosterol [21]. Among the fatty acids which are present in propolis, 10-hydroxy-Δ-2-decenoic acid, originating from the bees, deserves attention [4]. In the lipid-wax fraction of propolis saturated hydrocarbons like heptadecane, octadecane, eicosane, tricosane are present, as well as unsaturated hydrocabons: cholestrilene and eicosene [4,13]. Propolis contains a large amount of beeswax, which is composed of esters of higher alcohols and lipid acids, as well as of aliphatic hydrocarbons. The main components of beeswax are myricyl esters of palmitic and cerotic acids, cerotic and melissic acid. Beeswax also contains small amounts of alcohols, lactones, carotenoids, cholesterol esters and flavonoids. Among the flavonoids present in beeswax there is chrysin, which gives this product its specific colour [13,15].

Among other components present in propolis there are micro- and macroelements. About 30 elements have been discovered in propolis. Calcium, manganese, zinc, copper, silicon, iron and aluminium are present in the greatest amounts [4,22]. Also B-group vitamins have been found in propolis, together with vitamins C, D, PP and E, as well as provitamin A (β-carotene). Small amounts of enzymes have also been isolated: α-amylase, β-amylase, α-lactamase, β-lactamase, maltase, esterase and transhydrogenase [10,14]. The presence of enzymes is connected with the bee glandular secretion added to propolis. It is also possible that the enzymes come from pollen [1]. The total protein content in EEP amounts to 2.8%, on average [1]. Free amino acids are present—17 of them—but their content is low [4]. In recent years pyroglutamic acid—a bee organism amino acid—has been identified in propolis. Polysaccharides are also present there—starch, as well as di- and monosaccharides: saccharose, glucose, fructose, rhamnose, ribose, talose and gulose [13].

The most important components of Polish propolis with respect to both quantity and type are polyphenols, including phenolic acids and flavonoids. These compounds display powerful antioxidative properties and high biological activity. The antioxidative activity of polyphenols depends on their structure [11,14].

### 2.1. Phenolic Acids

**Figure 1:**
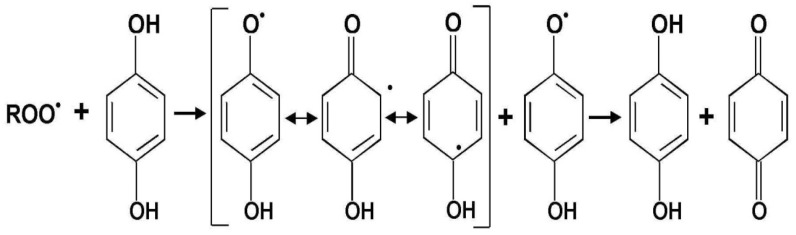
Oxidation of phenolic compounds [25].

Phenolic acids are compounds built of a benzene ring, carboxyl and hydroxyl groups. The antioxidative activity of phenolic acids depends on the number of hydroxyl groups in their molecules and on the steric effects [23]. The position of hydroxyl groups, as well as the type of substitution on the aromatic ring, influence the antioxidative activity of these compounds. This is result of fact that the energy of the bond between the atom of hydrogen and the atom of oxygen in the hydroxyl group attached to the aromatic ring system is smaller than in the case of aliphatic compounds. Decreased density of electrons in the atom of oxygen is a result of the resonance effect of the aromatic ring, because of which the atom of hydrogen is detached from the hydroxyl group and phenolic compounds become phenoxyl radicals, which are relatively stable because of the shift of charges in the aromatic ring (Figure 1). Joining, phenoxyl radicals create quinones or they enter into other reactions, such as dimerization or radical substitution [24,25].

The antioxidative activity of phenolic acids is inversely proportional to the size of the enthalpy of the dissociation of the O–H bond in a weakly polar environment. The mechanism of severing the O–H bond in a phenolic ring consists in hydrogen atom transfer (HAT) to a superoxide radical [26]. Monohydroxy derivatives of benzoic acid with one hydroxyl group in the *ortho*- or *para*-position in the ring do not display antioxidative activity, contrary to *meta*-monohydroxy derivatives of radicals generated in the aqueous phase. This is connected with the ability of the carboxyl group to attract electrons and influence the *ortho*- or *para*- position in the ring. Monohydroxy acids are effective hydroxyl radical scavengers. Furthermore, antioxidative activity increases in acids with one hydroxyl group together with the presence of additional methoxy group in the ring. The substitution of an alkyl or methoxy group in the *ortho*-position increases the stability and the antioxidant properties of phenolic acids [27].

Benzoic acid dihydroxy derivatives display the most powerful antioxidative properties when the hydroxyl groups appear in the positions 3 and 5. Among benzoic acid derivatives, gallic acid, containing three hydroxyl groups in the 3, 4 and 5 positions, is characterized by very good antioxidative properties [24]. Esterification of the carboxyl group in gallic acid decreases its antioxidant abilities. 

The influence of additional hydroxyl groups in the aromatic ring on the increase of antioxidative activity may be observed also in the case of cinnamic acid. Caffeic acid has a more powerful antioxidative effect than coumaric acid [28,29]. 

The power of antioxidative activity of phenolic acids depends on the location of the carboxyl group. The group has a negative impact on the donor properties of hydroxybenzoate and its derivatives. Having a side chain containing an ethylene group, hydroxycinnamic acids display greater ability to donate hydrogen and the radical created in this way is more stable. Thus, the derivatives of cinnamic acid display better antioxidative properties than benzoic acid derivatives [23,30]. The introduction of an ethylene group between the phenyl ring containing a hydroxy group in the *para*-position and a carboxyl group, as in *p*-coumaric acid, results in increased reductive properties of the hydroxyl group in comparison with cinnamic acid. Ferulic acid, with a methoxy group in the 3 position displays greater ability to stabilize phenoxy radicals than *p*-coumaric acid [24]. However, substituting hydrogen in the hydroxyl group with methyl group may have varied effects on the antioxidative activity of phenolic acids, depending on the polarity of the environment. In the lipophilic phase, dihydroxy- cinnamic acid derivatives display a greater ability to immobilize free radicals than monohydroxy derivatives. Caffeic acid is characterized by better reactivity towards radicals in a water environment than ferulic acid. Ferulic acid displays affinity to lipids, which is related to the substitution of the hydrogen atom in the hydroxyl group with a methyl group. Therefore, ferulic acid shows better antioxidative properties in emulsion type environments, such as oil in water, in comparison with caffeic acid [24]. Because of the presence of the methoxy group ferulic acid is more effective than *p*-coumaric acid. The methoxy group, as a donor of electrons, causes the increase in the ability to stabilize aryloxy radicals. Hydroxylation replaced with metoxylation makes a compound of such structure a more effective antioxidant. In their research on fats oxidation, Rice-Evans *et al.* determined that antioxidative activity varies in the following order: caffeic acid > ferulic acid > *p*-coumaric acid [24]. Brand-Williams *et al.* [31] demonstrated that ferulic acid neutralizes free radicals more effectively than BHA and BHT, while Gulcin proved that caffeic acid had more powerful protective properties than BHT [32]. The antioxidative activity of phenolic acids consists in “scavenging” free radicals (superoxide, hydroxyl, and hydroxyl superoxide ones), chelating ions of metals (iron, copper), as well as changing the activity of enzymes by inhibiting oxidases [33,34,35].

### 2.2. Flavonoids

**Figure 2:**
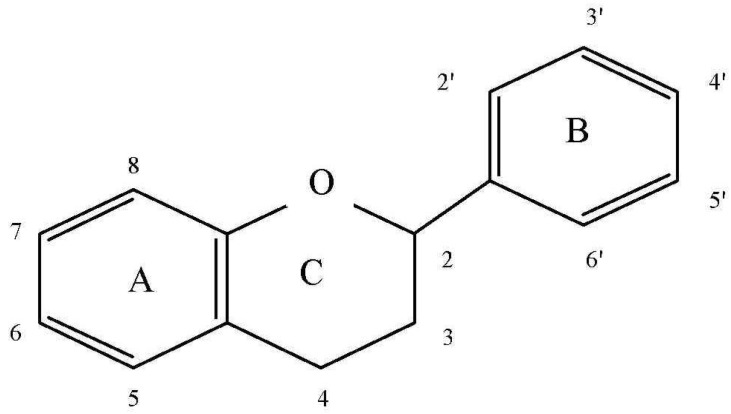
Basic flavonoid structure [39].

Flavonoids (Figure 2) are a numerous group of polyphenols, which vary widely with respect to structure and properties. They are the most widespread substances of plant origin [36,37]. The basic element of their structure is a C6-C3-C6 unit consisting of 15 carbon atoms, which includes a benzoic ring and a phenylpropane unit [38].

Most flavonoids create a heterocyclic system containing oxygen between their aromatic rings. Therefore, these compounds are considered derivatives of benzo-γ-pyrone (chromone). A frequent element of their structure is a double bond in the position C-2 and C-3, as well as the presence of a carbonyl group in the C-4 position [40,41].

Particular compounds differ from one another primarily as to the number and location of hydroxyl groups, which is crucial for their pharmacological properties. Most flavonoids contain a hydroxyl group at positions C-5 and C-7, which is in the A ring, while differences in hydroxylation appear mainly in the phenylpropane unit, which usually has a catechol structure in the B ring, with OH groups at positions 3' and 4' [39]. However, sometimes compounds occur which have additional or differently located hydroxyl groups in the benzoic ring. Further differentiation consists in the varied extent of the presence or absence of an unsaturated 2–3 bond in conjugation with a 4-keto group in ring C, the presence of methoxy groups, the type of glycosidic bond with monosaccharides, sugar acids or other organic acids, as well as in the presence of a dimeric unit—the so-called biflavonoids—or polymer units of the basic 15-carbon skeleton [38]. In plants these compounds usually appear as glycoside structures, in which the sugar fragment contains 1–5 molecules of monosaccharides linked with O- or C-glycoside bonds. Among the most frequently appearing sugars are hexoses (glucose, galactose), pentoses (arabinose, xylose, mannose) or uronic acids (glucuronic acid, galacturonic acid); these are often esterified with sulphuric acid, aliphatic (acetic, malonic acid) or aromatic acids (benzoic, cinnamic). If the chromone is substituted with three or more sugars it may lead to further differentiation due to the appearance of different branching patterns. Sugar radicals are present in O-glycosides primarily at positions C-3, C-5, C-7, C-3' and C-4', while in C-glycosides they are at the C-6 and C-8 positions [42]. Sugar residues are hydrolyzed in the alimentary tract by means of enzymes produced by the bacteria of the digestive system [43]. The basic classes of flavonoids are: flavones, flavonols, flavanones, flavanonols, isoflavones, flavan-3-ols (catechins), chalcones, anthocyanidins, and leukoanthocyanidins [38,44].

Flavonoids are characterized by powerful antioxidative properties. These properties are closely related to the structure of these compounds. The effective antioxidative activity of flavonoids results from the following elements of their structure [24,26,39,45,46,47]:

- the *ortho*-dihydroxy (catechol) group in B ring, which displays significant ability to “scavenge” oxygen (ROS) and nitrogen radicals (RNS) and ensures the high stability of the created phenoxyl radical. Hydroxyl groups in B ring are donors of electrons and nitrogen for radicals.
- the double bond between C-2 and C-3 carbon and the presence of the 4-oxo group in the C ring is the reason for the dislocation of an electron in the B ring. The antioxidative properties result from the dislocation of an electron of the aromatic unit. During the reaction of the compounds with free radicals, phenoxyl radicals are created and stabilized by the effect of the aromatic ring resonance.
- hydroxyl groups near C-3 and C-5 carbon in the presence of 4-oxo groups in the A and C ring generate the maximum free radical scavenging effects.

Glycosylation at position C-3 decreases the antioxidative abilities of flavonoids [45,48]. Aglycones are more powerful antioxidants than their glycosidic linked forms. Apart from the presence and number of sugars itself, their position and structure play a significant role [39]. The antioxidative properties are also decreased by the presence of methoxyl groups in the C-3 position in the flavonoid, probably as a result of steric hindrance [39,47,48,49]. The most powerful antioxidative properties are displayed by flavon-3-ols (e.g., -quercetin, myrycetin, morin), flavan-3-ols—catechins (epicatechin gallate, epigallocatechin gallate, epigallocatechin, catechin) anthocyanidins (cyanidin) [24,39].

## 3. The Biological Properties of Polyphenols, Including Their Antioxidative Activity

The antioxidative activity of polyphenols is one of their most appreciated properties. A wide spectrum of biological activity of these compounds with respect to the human body largely results from their antioxidative effects [26,50]. The sources of free radicals in the organism are most often reactive forms of oxygen (ROS). They are created as a result of gradual reduction of molecular oxygen in one-electron reactions. Reactive forms of nitrogen (RNS)—nitrogen oxide NO^●^, nitrogen dioxide NO_2_^●^ and nitric acid HONO_2_—are also dangerous for health. Organic radicals formed as a result of ROS and RNS reacting with organic molecules are just as harmful. 

Unsettling of the balance between production and deactivation of ROS leads to many disorders [41]. Free radicals are able to oxidize cell proteins, nucleic acids and lipids. They contribute to aging of cell proteins, mutagenesis, carcinogenesis, the development of diseases such as Parkinson’s or Alzheimer’s. They also increase the risk of cardiovascular diseases, probably by destabilizing cell membranes and by LDL lipoprotein oxidation [39,46].

Polyphenol compounds are natural exogenous antioxidants. Ingested with food, they are absorbed unchanged, or they are metabolized by means of hydroxylation, methylation, sulfation and glucuronidation [36,43,47]. The bioavailability of polyphenols depends on the type of food ingested. Proteins bonding with them decrease their absorption, while alcohol increases it. Consumed polyphenols are mostly degraded by the intestinal bacterial microflora. A small proportion of them are absorbed in the form of aglycons and glycoside forms, the rest are degraded to various phenolic acids depending on the structure of the original flavonoid [45]. The metabolites created have an unchanged structure of units responsible for their antioxidative properties, so they display similar antioxidative activity to the unchanged compounds [47]. The mechanism of the antioxidative activity of polyphenols consists of [36,43,45,46]:

- inhibiting the activity of enzymes and thus inhibiting the appearance of reactive forms of oxygen (ROS)
- chelating ions of metals involved in the process of free radical creation
- scavenging reactive forms of oxygen (ROS), thus interrupting the cascade of reactions leading to the peroxidation of lipids
- synergistic action with other antioxidants

Polyphenol compounds have the ability to inhibit the activity of enzymes which participate in the creation of reactive forms of oxygen. They decrease the activity of xanthine oxidase, an oxidase of reduced nicotinamide adenine dinucleotide phosphate (NADPH), responsible for the appearance of superoxide anion radical [43,45,51]. They also inhibit other enzymes, including, among others, protein kinase C, ascorbic acid oxidase, cyclooxygenase (COX-1 and COX-2), lipoxygenase (5-LOX, 12-LOX, 15-LOX), Na^+^/K^+^ ATPase, cAMP phosphodiestrase [37,45].

**Figure 3:**
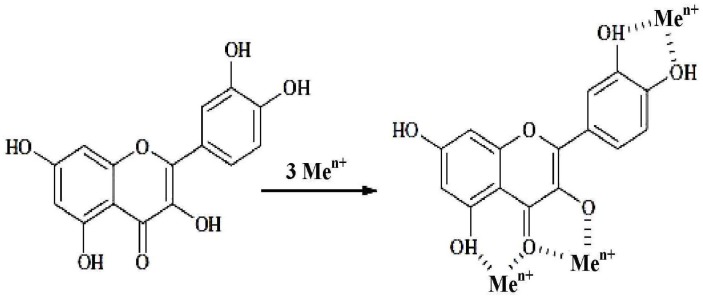
Metal-binding sites for flavonoids [26].

Many flavonoid compounds effectively chelate transition metal ions, mainly iron and copper, which, apart from performing physiological functions in the organism (they are, among others, key cofactors of enzymes and components of proteins) perform a crucial role in oxygen metabolism [37,45,51]. They catalyze the reduction of H_2_O_2_, during which a very reactive hydroxyl radical OH^●^ is created [37,52]. In the Fenton reaction, which is an important source of RFT in the case of the presence of a large amount of iron or copper ions, flavonoids with 4-oxo units, catechol units and hydroxyl groups in the C-3 and C-5 position are the most reactive (Figure 3) [26].

Flavonoids also inhibit free radical lipid oxidation reactions, in which copper, acting as a catalyst, allows for LDL lipoprotein oxidation [26,37,45]:

LH → L^●^ → LOO^●^, LH (LDL) [42]

Chelation is also the most frequent mechanism of enzyme inactivation [37].

Flavonoids and phenolic acids, due to their low redox potential, are thermodynamically able to react with highly reactive forms of oxygen by donating a hydrogen atom [26,37,45,47]:

Fl-OH + R^●^ → FL-O^●^ + RH

where R^●^—superoxide anion radical, hydroxyl radical, alkoxy radical, lipid radicals 

Lowering the level of ROS, polyphenols contribute to lipid peroxidation [39,45,46,47]. Also, the lipophilicity of these compounds, which allows them to penetrate lipid bilayers, enables them to inhibit peroxidation [46]. Flavonoids can also, by direct influence, stabilize biological membranes, making them more resistant to oxidation by decreasing their permeability [47].

Non-enzymatic peroxidation may be halted at the stage of:

- initiation, as a result of the inactivation of the radical initiating the process (hydroxyl radical):

FL-OH + OH^●^ → H_2_O + FL-O^●^

- propagation due to the inactivation of lipid superoxide radical (LOO^●^):

LOO^●^ + FL-OH → LOOH + FL-O^●^

- termination as a result of the inactivation of lipid peroxide radical (LOO^●^), lipid radical (L^●^) and alkoxy radical (LO^●^), appearing as a result of lipid peroxidation by metal ions:

LOO^●^/LO^●^/L^●^ + FL-OH → LOOH/LOH/LH + FL-O^●^

FL-OH—flavonoids
FL-O●—phenyloxyl radical [47]

The resulting radical is less aggressive and may undergo further radical reactions to give a stable structure [45]. In the case of scavenging singlet oxygen and superoxide anions, radical flavonoids are transformed into stable products [47]. 

Flavonoids decrease the activity of enzymes participating in enzymatic peroxidation, for example, phospholipase A2 (PLA2), which is one of the enzymes initiating the peroxidation process [47]. 

Polyphenolic compounds have a protective influence on endogenic antioxidants such as ascorbic acid or tocopherol by chelating metals which catalyze their oxidation reactions [47]. Some of them have the ability to stabilize pro-oxidative α-tocopherol radicals. The protective activity is mutual. Furthermore, polyphenols increase the absorption of ascorbate, stabilize its particles, reduce its oxygenated form and decrease its metabolism [43]. Because of its high content of various polyphenol compounds, propolis displays powerful antioxidative activity, which has been confirmed in many studies, both *in vitro* [52,53,54,55] and *in vivo* [56].

### 3.1. Antibacterial Activity

The antibacterial properties of propolis have been proven in many studies, where much greater activity was determined with respect to Gram positive bacteria than to Gram negative ones [57]. Propolis also has a powerful antibacterial influence on *Staphylococcus aureus* strains and bacteria of this type are most often used to assess propolis activity [58].

Studies have proven the high activity of propolis with respect to *Mycobactrium tuberculosis*, *Mycobacterium avium*, *Staphylococcus epidermidis*, *Streptococcus piogenes* and *Klebsiella pneumoniae* [59]. In the scientific experiments conducted, propolis displayed synergistic activity to that of several antituberculotic drugs (streptomycin, rifamycin, isoniazid) and other antibiotics (for example, chloramphenicol, gentamicin, vancomycin, tetracycline, clindamycin, netilmicin) [60].

The antibacterial properties of propolis may be the result of the synergistic activity of the many compounds present in propolis. Pinocembrin displays an intense antibacterial activity against *Streptococcus sp*. Apigenin most powerfully inhibited bacterial glycosyltransferase. *p*-Coumaric acid, artepillin C and 3-phenyl-4-dihydrocinnamylocinnamic acid were effective against *Helicobacter pylori* [61]. In experimental observations concerning bacterial flora developing in burn wounds, the therapeutic influence of Polish propolis was demonstrated, manifested by a lack of pathogenic flora development and greater effectiveness than 1% silver sulfadiazine (SSD) [62]. The therapeutic effectiveness of Polish propolis extract against *Staphylococcus aureus*, isolated as a strain causing nosocomial infections, was demonstrated in *in vitro* studies. Propolis displayed bactericidal properties, despite the fact that the studied strain displayed great antibiotic resistance with respect to many existing chemotherapeutics [63]. Clinical studies demonstrated therapeutic and preventive effectiveness of propolis with respect to bacterial flora on the skin of patients suffering from para- and tetraplegia, which contributes to the process of bed sore generation [64].

### 3.2. Anti-Inflammatory Activity

The anti-inflammatory activity of phenolic acids and flavonoids is a result of their antioxidative properties [52,65]. When comparing the chemical structure of flavonoids displaying anti-inflammatory activity, it has been demonstrated that these properties are determined by C-3' and C-4' hydroxyl groups in the B ring, of which there cannot be more than two [37].

Anti-inflammatory activity is connected with the decreased synthesis of prostaglandin E2 (PGE2), thromboxane A2, leukotriene B4 and NO (nitric oxide II), which participate in inflammatory reactions. It is the effect of inhibiting a cascade of arachidonic acid transformations, due to the inhibiting influence on phospholipase A2, lipoxygenase (5-, 12-, 15-), cyclooxygenase (COX-2) as well as nitric oxide synthase (iNOS). This activity is comparable with the analgesic action of indometacin [44,66,67,68]. 

Because of its large content of polyphenol compounds, propolis displays anti-inflammatory activity both in acute and chronic inflammatory processes [69]. propolis has a significant effect on the arachidonic acid metabolic pathway. In experimental studies the inhibiting activity of propolis extract with respect to cyclooxygenase COX-1 and COX-2 [70] and lipoxygenase activity [71] was documented. The effects of propolis extract activity are changes in the concentration of prostaglandin E2 and leukotrienes. The powerful inhibiting activity of caffeic acid phenethyl ester (CAPE) on the enzymes of arachidonic acid metabolic tract and weak galangin effect on cyclooxygenase activity were demonstrated [70,71]. 

Interleukin IL1β has a great impact on the appearance and spread of inflammatory reactions. The inhibiting influence of propolis on its synthesis through the inhibition of mRNA IL1β and synthase NO expression was shown [72,73]. Chrysin, kaempferol, quercetin and galangin present in propolis influence mRNA expression—the greatest activity has been observed in the case of quercetin [72]. 

Studies have demonstrated the inhibiting activity of propolis with respect to NADPH oxidase, ornithine decarboxylase, myeloperoxidase and hyaluronidase [74]. 

A specific effect of propolis in the form of platelets aggregation inhibition and prostaglandin and 5-lipooxygenase (5-LOX) synthesis [75] has been experimentally proven. It has also been proven that the anti-inflammatory effect of propolis is the same as that of non-steroidal anti-inflammatory drugs, yet free from the side effects of those drugs [76]. 

In experimental studies conducted on rats based on an arthritis model, anti-inflammatory effects of propolis were demonstrated, achieved due to the prostaglandin inhibition effect [77]. Propolis extracts cause a non-specific immunological response through macrophage activation and the inhibition of nitric oxide NO [78].

Studies on propolis water extracts have shown that they have a suppressive effect on cell migration. This effect may be used to control the inflammatory response without deteriorating the conditions for cell repair processes. This phenomenon is a result of a high content of caffeic acid [79]. Propolis extracts also contribute to the improvement of the activity of hepatic enzymes, lipid profile parameters and bilirubin content in the case of inflammation and toxic liver damage. The effect was manifested by the lowered level of IL-6, TNF-α and CRP [80].

### 3.3. Anticarcinogenic Effects

Propolis contains biologically active substances which are known to be promoters that stimulate cell proliferation or apoptosis. Among them there are caffeic acid, caffeic phenyl ester, artepillin C, quercetin, naringenin, resveratrol, galangin, and genistein [81]. Flavonoids consumed with food have a direct effect on cell proliferation, differentiation and the apoptosis of cancer cells, especially with respect to gastrointestinal tract cancer because of their direct contact with ingesta. The direct cytotoxic effect of flavonoids contained in propolis is significant in the case of breast cancer and papillomatous urinary tract tumours [82]. Flavonoids present in propolis stop the proliferation of various types of cancer cells, especially monocytic and lymphatic leukemia [83]. The anticancerogenic mechanism consists in the inhibition of tyrosine kinase C, which participates in the growth and proliferation of cancer cells [84].

Genistein, quercetin, kaempferol, myricetin, luteolin, chrysin and apigenin inhibit cyclin D1 and cyclin E, by which they arrest the cell cycle [85]. Caffeic acid analogues are effective blockers of the prooxidative enzyme which is xanthine oxidase [86]. Galangin, genistein, hesperitin, naringenin and resveratrol display antiproliferative activity with respect to the breast cancer estrogen receptor [87]. In tests conducted on animal experimental models it has been demonstrated that flavonoids contained in propolis inhibit the development of lung cancer and oral cancer, as well as skin, esophagus, stomach, colorectal, prostate and breast cancers [88].

In 1998 Kimoto *et al.* demonstrated that artepilin C present in propolis displays cytostatic and cytotoxic effect with respect to cancer cells through immunostimulation by activating macrophages, especially by increasing their phagocytic activity [89]. Slavin *et al.* showed that hydroxycinnamic acids—ferulic and caffeic acid—inhibit cancer development and the appearance of mutagenic nitrosamines [90].

### 3.4. Antiatherogenic Effects

Flavonoids also display antiaggregative effects on blood platelets. This consists in the possibility to break the chain reaction of lipid oxidation initiated by free radicals. It has been proven that superoxide anions shorten the half-life period of endothelium-derived relaxing factor (EDRF), which is characterized by antiaggregative and relaxing effects on blood vessels. Hydroxyl free radicals also inhibit prostacyclin synthetase responsible for the transformation of prostaglandin peroxides into prostacyclin, which acts similarly to EDRF. Apart from the ability to reduce the production of free radicals and the antiaggregative activity flavonoids must also have the possibility to bond with blood platelets [91,92].

As a conglomerate of phenolic acids and flavonoids, propolis is a modulator of lipid and lipoprotein metabolism and has direct influence on the lowering of the cholesterol levels and triglyceride synthesis in the liver of laboratory rats [93,94]. In mice with deactivated LDL receptor treated with propolis extract, a lowering of total cholesterol triglyceride level was noted [95]. 

In the case of toxic damage to mouse organisms caused by administering alcohol, with simultaneous propolis extract therapy a therapeutic effect was obtained in the form of the improvement in lipid profile parameters. LDL fraction was lowered and, at the same time, an increase in HDL was observed [96]. 

Based on the most recent studies it was determined that propolis displays preventive effects with respect to atherosclerosis. By administering propolis an improvement in lipid profile and decrease in the content of pro-inflammatory cytokines and chemokines was achieved. The effect was a result of influencing mRNA by regulating the expression of genes which play a key role in the atherosclerosis pathomechanism, such as MCP-1 [95]. The proposed mechanism regulating the influence of propolis on lipid metabolism is its direct influence on the expression of ABCA1 gene, which causes the increase in the HDL fraction level [97]. 

Aggregation of blood platelets is one of the components in the development of atherosclerotic lesions. CAPE contained in propolis is an inhibitor of platelet aggregation [98]. Propolis extracts also have a direct effect on the synthesis of nitric oxide. The modulating influence on the level of nitric oxide synthesis has a protective effect on the blood vessel endothelium in the course of cardiovascular inflammatory processes [99].

### 3.5. Effects on the Cardiovascular System

Polyphenol compounds, mainly flavonoids, stabilize and strengthen blood vessels. Therefore, they may be used in the prevention of bleeding, ecchymoses, varicose veins and atherosclerosis [36]. Vasoprotective activity is connected with chelating copper ions, which results in inhibiting the hyaluronidase which depolimerises hyaluronic acid, and the hyaluronidase which hydrolyzes elastin, thus strengthening and sealing vessel endothelium [41]. Vessel permeability is also decreased indirectly in effect of inhibiting the metabolism of catecholamines by inactivating catechol-*O*-methyltransferase. This activity is defined as type P vitamin activity [37].

Polyphenols also display beneficial influence on coronary circulation and have hypotensive effect, which probably results from their similarity to β-blockers in chemical structure [38]. They increase the bioavailability of nitric oxide by enhancing the activity of endothelial nitric oxide synthase (eNOS). They also inhibit angiotensin convertase enzyme (ACE), by exerting vasodilator effect. They decrease blood platelet aggregation and the concentration of thromboxane [100,101].

Tests have shown that puerarin and daidzein act on blood vessels with power equal as nitroglycerin. These properties are an effect of the flavonoids’ inhibiting influence on the activity of angiotensin-converting enzyme (ACE) and enzyme converting phosphodiestrase cAMP (cyclic 3'5'-adenosino-triphosphate) as well as cyclooxygenase, which is connected with effects on vascular resistance and blood platelet aggregation. Scientists demonstrated that quercetin, troxerutin and rutin inhibit blood platelet aggregation more effectively than acetylsalicylic acid in a similar dose. Flavonoids display beneficial effects on vessel endothelium and cardiac muscle also because of the fact that they inhibit xanthine oxidase in these structures. This results in the decrease of superoxide and hydroxide free radicals creation during ischaemia [39,52,92]. 

Flavonoids contained in propolis such as quercetin, kaempferol and rhamnetin block the transport of calcium through cell membranes to the cytoplasm, which causes vessel dilation and decreases in blood pressure. Thanks to the aforementioned properties, propolis is used in the prevention of circulatory system diseases [102]. 

The protective properties of propolis with respect to the cardiovascular system result from its antihypertensive activity, which was proved on a rat animal model [103]. Cardioprotective effects were also achieved in the case of induced experimental cardiomyopathy in rats, which were treated by intraperitoneal administration of propolis extract. Improvement both in biochemical parameters and in the histological image of the cardiac muscle was observed [104].

### 3.6. Estrogenic Effects

Because of the structural similarity of flavonoids and isoflavonoids to the endogenic sex hormones there is an increased interest among scientists in the estrogenic effects of propolis. Flavonoids demonstrate affinity to ER-α estrogen receptors present in the breast, endometrium and ovaries, as well as ER-β estrogen receptors present in the brain, blood vessels, lungs and bones. The potential estrogenic activity of ethanol and ether extracts of propolis was studied by Song *et al.* The results of these studies show that propolis displays estrogenic activity by activating estrogen receptors [105]. Jung *et al.* demonstrated that caffeic acid phenethyl ester (CAPE), the structure of which resembles phenolic acids, is responsible for, among others, the estrogenic effect of propolis. CAPE displays more affinity to ER-β receptor than to ER-α receptor. Studies show that CAPE is a selective agonist of ER-β and a potential modulator of the estrogen receptor [106].

Further studies are necessary on the influence of bioactive components of propolis on the estrogen receptor. Some researchers have observed that ingesting propolis containing chrysin blocks the transformation of androgens into estrogens by inhibiting aromatase, which causes an increase in the level of testosterone. Flavonoids such as chrysin and galangin, as well as flavones such as naringenin, decrease estrogen biosynthesis by inhibiting aromatase. Similar activity was observed in the case of apigenin [107,108].

### 3.7. Antidiabetic Effects

The occurrence of noninsulin-dependent diabetes mellitus (NIDDM) in the populations of the developed countries is steadily increasing. Therefore, research is being incessantly conducted into natural substances which may contribute to lowering postprandial glucose. Tests conducted *in vitro* and *in vivo* demonstrate that flavonoids may have antidiabetic effects. Epicatechin stimulates insulin synthesis and increases the level of cAMP in β cells of Lagerhans islets, increasing insuline secretion. EGCG (epigallocatechin 3-gallate) has hypoglycemic effect by inhibiting the production of glucose in the liver. Flavonoids may also influence glucose absorption in the intestine. Daidzein, luteolin, and the 7-*O*-glucoside of luteolin inhibit the activity of α-amylase and β-glucosidase and quercetin glycosides influence SGLT-1 glucose transporters in enterocytes [109].

Flavonoids seem precious natural compounds not only because they prevent rapid blood sugar rises in the serum, but also because they can protect diabetics from the complications of this metabolitic disorder. It has been demonstrated that quercetin prevents the development of cataracts in diabetics. Quercetin also inhibits aldose reductase, which participates in the synthesis of sorbitol deposition in the eyeball, which causes the development of cataracts [110]. 

Studies by Matsui *et al.* conducted on Brazilian propolis demonstrated antihyperglycemic effects with respect to caffeoylquinic acids (CQA). Caffeoylquinic acids are powerful inhibitors of β-glucosidase and α-amylase. CQA family including caffeic acid, 3-caffeoylquinic acid (chlorogenic acid), 3,4-di-caffeoylquinic acid, 3,5-di-caffeoylquinic acid and 3,4,5-tri-caffeoylquinic acid displays antihyperglycemic effects through the inhibition of intestinal maltase activity. 3,4,5-tri-Caffeoylquinic acid displays the most powerful effect and has a potential to be used to achieve specific retardation of maltase activity [111].

Fuliang *et al.* demonstrated in their research, conducted on rats with diabetes, that the administration of propolis extracts leads to lowering of the levels of glucose, fructosamine, malonic aldehyde, nitric oxide, nitric oxide synthetase, total cholesterol and LDL fraction, which suggests its protective activity by lowering the level of lipid peroxidation [112].

### 3.8. Anti-HIV Activity

Researchers have proven the antiviral properties of flavonoids so the compounds are considered potential therapeutic anti-HIV agents. Flavonoid antiviral activity was comprehensively discussed in the studies of Wang *et al.* [113]. Flavonoids act as reverse-transcriptase inhibitors—reverse transcriptase is an enzyme necessary for the development of HIV—as well as inhibitors of RNA-directed DNA polymerase [114]. Middleton *et al.* also described the activity of antiintegrase and antiprotease [115]. Active propolis components, such as caffeic acid phenethyl ester, quercetin and kaempferol, disturb the replication of HIV-1 by inhibiting HIV-1 integrase. Veljkovic *et al.* have shown that epicatechin, baikalin and EGCG inhibit the penetration of the virus into the cell as a result of disturbing the interaction between the proteins in viral envelope and the attacked cell surface molecules. Furthermore, quercetin inhibits the activity of viral Vpr protein, which is responsible for the proliferation of the virus and activates integrase and proteinase [116]. 

Studies conducted on Brazilian propolis have demonstrated that it displays a significant anti-HIV activity. Ito *et al.* [117] have demonstrated that moronic acid present in Brazilian propolis is characterized by anti-HIV activity EC50 < 0.1 μg/mL. It has been observed that the modification of betulic-acid-related and oleanolic-acid-releated triterpenoids increases the potential effects of these compounds on HIV virus. 3β-Hydroxy group esterification in these two triterpenes increases the anti-HIV activity [117]. Considering the above data, biologically active components of propolis may become in future potential therapeutic agents in AIDS treatment.

### 3.9. Reparative-Regenerative Effects

A highly significant biotic function of propolis is its reparative-regenerative effect on tissue defects [118]. Standardized propolis extracts display regenerative effects on damaged tissue. They owe this to the fact that they contain many compounds which comprehensively act to enhance cell and systemic metabolism by activating ATPase and tetrasol reductase. This increases mitotic index and by that facilitates tissue regeneration and shortens the healing period [119].

The synergistic activity of phenolic components leads to modulating the accumulation of type I and III collagen in the place of thermal tissue damage. Owing to this, biochemically conducive conditions are created for the healing processes to take place, minimizing excessive scarification or the appearance of keloids [120]. In laboratory studies the influence of propolis was demonstrated onto the accumulation of chondroitin and hyaluronic acid, which to a large extent influences the effectiveness of the remodelling phase of the reparative process [121].

Clinical studies conducted by many centres have demonstrated the high therapeutic effectiveness of propolis extracts when used in bedsore prevention and therapy. In 85% of patients the effectiveness of propolis preparations in bedsore prevention was demosntrated [64].

In experimental studies conducted on an animal model it was shown that propolis extracts incorporated in various vehicles (gels, ointments) display a great therapeutic potential with respect to third degree burn wounds. Test results and clinical observations were confirmed by the histopathological assessment of tissue samples. The preparations were more effective therapeutically than silver sulfadiazine [122].

The regenerative activity of ethanol extracts of Polish propolis has been proven also with respect to animal endodontium [123] and in the case of articular cartilage in dogs [124]. In 1998 Buczek *et al.* described a case of postoperative gas oedema in the course of proximal tibia fracture. In therapy employing surgical procedures propolis ointment based on Polish propolis extract was used. Full therapeutic effect was achieved without the need to amputate the affected limb [125].

## 4. Conclusions

As a substance of natural origin, propolis does not have a stable and reproducible chemical composition. Thus, a serious research problem arises, that of defining its full composition in a credible way. Despite its variety, it is always highly biologically active. Among the most significant chemical compounds comprising Polish propolis are phenolic acids and flavonoids. Owing to their structure, these compounds display high antioxidative activity. Tests conducted on polyphenols present in Polish propolis have allowed scientists to establish the mechanism of polyphenol activity and proved their antibacterial, anti-inflammatory, anticarcinogenic, antiatherogenic and reparative-regenerative activity. In consideration of the high antioxidative activity of Polish propolis new therapeutic possibilities connected with this natural bee product are being actively sought. 
